# Implementation of respondent driven sampling in Nairobi, Kenya, for tracking key family planning indicators among adolescents and youth: lessons learnt

**DOI:** 10.1186/s13104-022-06038-8

**Published:** 2022-06-07

**Authors:** Mary Thiongo, Peter Gichangi, Patrick K. Macho, Meagan E. Byrne, Peter Kimani, Michael Waithaka, Scott Radloff, Philip Anglewicz, Michele R. Decker

**Affiliations:** 1 International Centre for Reproductive Health, Mombasa, Kenya; 2 Technical University of Mombasa, Mombasa, Kenya; 3 Department of Public Health and Primary Care, Faculty of Medicine and Health Sciences, Ghent University, Ghent, Belgium; 4 School of Public Health, College of Health Sciences, Moi University, Eldoret, Kenya; 5 Department of Population, Family and Reproductive Health, Johns Hopkins Bloomberg School of Public Health, Baltimore, MD USA; 6 Present Address: P.O. Box, Nairobi, 2631-00202 Kenya

**Keywords:** Adolescents, Contraception, Family Planning, Methodology, Respondent-Driven Sampling, Youth, Nairobi, Kenya

## Abstract

**Objective:**

Adolescents and youth constitute a significant proportion of the population in developing nations. Conventional survey methods risk missing adolescents/youth because their family planning/contraception (FP/C) behavior is hidden. Respondent-driven sampling (RDS), a modified chain-referral recruitment sampling approach, was used to reach unmarried adolescents/youth aged 15–24 in Nairobi, Kenya to measure key FP/C indicators. Seeds were selected and issued with three coupons which they used to invite their peers, male or female, to participate in the study. Referred participants were also given coupons to invite others till sample size was achieved. We report on key implementation parameters following standard RDS reporting recommendations.

**Results:**

A total of 1674 coupons were issued to generate a sample size of 1354. Coupon return rate was 82.7%. Study participants self-administered most survey questions and missing data was low. Differential enrolment by gender was seen with 56.0% of females recruiting females while 44.0% of males recruited males. In about two months, it was possible to reach the desired sample size using RDS methodology. Implementation challenges included presentation of expired coupons, recruitment of ineligible participants and difficulty recruiting seeds and recruits from affluent neighborhoods. Challenges were consistent with RDS implementation in other settings and populations. RDS can complement standard surveillance/survey approaches, particularly for mobile populations like adolescents/youth.

**Supplementary Information:**

The online version contains supplementary material available at 10.1186/s13104-022-06038-8.

## Introduction

Use of modern contraceptives is an important public health intervention and a cost-effective strategy to reduce maternal mortality and avert unintended pregnancies. Globally, family planning/contraception (FP/C) programs have generated gains in contraceptive coverage particularly among married women of reproductive age (MWRA). Modern contraceptive prevalence among MWRA increased worldwide between 2000 and 2019 by 2.1 percentage points from 55.0 to 57.1% [1]. The coverage of contraceptives in Eastern Africa stands at 40% and is expected to grow to 55% by 2030 [2]. In Kenya, FP/C use increased among married women from 33% in 1993 to 53% in 2014 [3] and from 54% in 2014 to 61% in 2020 [4].

Despite overall improvement in FP/C use, adolescents and young women lag behind [3, 5]. An estimated 21 million young women aged 15–19 years in low-income and middle-income countries (LMICs) become pregnant every year; 12 million of whom give birth [6]. Most adolescent pregnancies and births are as a result of unplanned pregnancy; youth often lack knowledge and resources to make decisions about pregnancy planning and timing [7]. For young women, social and familial sanctions discourage and stigmatize sexual activity and contraceptive use [8]. Adolescent sexual reproductive health efforts must be contextualized and tailored to youth developmental needs for maximum impact [9, 10].

Evidence-based interventions for FP/C programs requires a robust monitoring system, yet youth can be a challenging population for surveillance with the standard methods that rely on household-based sampling and on-site data collection. The mobility of youth can render them under-represented through household-based recruitment. Under-reporting of key FP/C indicators can occur if youth are uncomfortable disclosing sexual risk behavior during home-based data collection, for example, pre-marital sexual activity that may be stigmatized or shamed, particularly for young women [8, 11–16].

Respondent-driven sampling (RDS) is a modified chain-referral recruitment sampling approach designed for hard-to-reach populations, i.e., those for which a sampling frame does not exist and/or acknowledgment of membership has potential consequences [17–19]. (RDS) has been explored as an alternate or supplemental approach [11] for reaching adolescents. However, implementation information for youth remains limited. RDS survey data can be weighted to compensate for its not having been drawn randomly [18, 20]; controlled, tracked recruitment supports analytic adjustment for non-independence among participants. RDS has been used to reach a range of hidden populations [11, 17, 21–23] for family planning [24, 25] and hidden behaviors [11, 17, 21, 23]. RDS is premised on the assumption that peers are better able than outreach workers and researchers to locate and recruit other members of the same population.

While several features of RDS make it appealing for research with youth, less is known about implementation to guide where and how it can supplement existing surveillance methods. In this paper, we discuss lessons learnt for RDS implementation among unmarried adolescents and youth in urban Nairobi.

## Main text

### Methods

#### Study design, setting and population

From June to August 2019, a cross-sectional survey among unmarried adolescent and youth aged 15 to 24 years in Nairobi, Kenya via RDS was done to measure contraception indicators. Detailed methodology is included as Additional material (Additional file 1).

#### Formative phase

Following RDS recommendations [26, 27], formative research activities included focus group discussions with youth group members and stakeholders affiliated with youth and family planning service provision to inform RDS acceptability, logistics, and survey scope. We assessed youths’ network properties including subgroupings and networking within and across subgroupings, identified necessary seed characteristics and potential seeds, clarified optimal recruitment field sites for confidentiality, access and comfort, and refined survey domains.

#### Seed selection and recruitment

Seeds are members of the target population who represent diversity with regard to underlying subpopulations in order to start the recruitment chains. Taking into consideration gender, age, marital status, level of schooling, and current school status (in-school or out-of-school) and subcounty in Nairobi, seven seeds were launched on June 21–22 (5 females, 2 males), and two male booster seeds were launched in mid-late July, for a total of nine seeds. Recruitment of the target sample size was achieved through peer-to-peer coupon distribution. Target sample size was 1300, which was calculated based population-based prevalence estimates from Nairobi, and adjusted for design effect and potential field recruitment error rate.

#### Data collection

Study enrolment and procedures took place at seven sites throughout Nairobi County to facilitate access to the study to youth in different neighbourhoods. The sites were operated by International Centre for Reproductive Health, Kenya (ICRHK) study staff in partnership with youth-friendly community-based organizations identified during the formative research phase. Study participation involved a one-time visit to any study site. To prevent duplicate participation, fingerprint scanning was implemented with fingerprint data stored separately from survey data. Study staff verified participant age eligibility using photo identification. Parental consent for minors under age 18 was waived by the KNH-UON Ethics and Review Committee but the minors gave written assent. Ethical approval to undertake the study was given by the KNH-UON Ethics review committee.

The survey focused on sexual and reproductive health. The survey was developed in English, professionally translated into Swahili, and piloted with native speakers to ensure comprehension. Trained research assistants (RA) obtained consent. Following informed written consent/assent, participants, males or females aged 15 to 24 years self-reported the size of their social network. To improve accuracy [26], network size. three questions. (1) How many youth between age 15 and 24 who are unmarried and live in Nairobi do you know personally (know their names)? (2) How many of those acquaintances (unmarried youth ages 15–24) also know you? Meaning they know your name and how to reach you, and (3) How many of them have you seen or spoken to at least once in the last six months? were asked sequentially by the RA, and structured to ensure reciprocity in social ties. To maximize confidentiality and minimize bias, the survey was self-administered via a handheld tablet, which has been used to enhance accuracy in reporting on sensitive topics [28]. Staff assistance and/or staff administration of the questionnaire was available in cases of limited literacy, difficulty comprehending the questions, or unfamiliarity with use of a tablet. If the participant opted to self-administer the questionnaire, a member of the study staff was always present in the room to answer questions. Participants could opt to take the survey in either English or Swahili language. All interviewers were fluent in both languages.

After survey completion, the seeds and subsequent recruits were provided with up to three recruitment coupons, with a short training on coupon distribution. Participants received a primary compensation of 500KES (approximately US$5) and US$5 for transport reimbursement, and a secondary compensation of 300KES (approximately US$3) per recruited eligible participant for up to three. All compensation was distributed using M-Pesa, a mobile phone-based money transfer system, so participants did not need to return to a study site to collect their secondary incentive.

#### Controlled recruitment via coupon management

Each coupon had an expiration date, generally 3–5 days after the validation date, after which it could not be redeemed. Coupon expiration dates were used to control recruitment pace and to end recruitment when the sample size was achieved. Coupons were identifiable by sequential numbers which linked recruits to their recruiters, enabling creation of recruitment chains. Coupon data were input into electronic coupon manager forms, which were uploaded and monitored daily for duplicate coupons and missing referral linkages. All coupons included a coupon number, barcode, addresses of the study sites, study hours, site phone numbers, and a description of study eligibility criteria. To taper participant enrolment, coupon distribution was reduced to one outgoing coupon per participant on July 18 and ended on July 29 for recruitment chains originating from seeds 1–8. Coupon distribution ended on August 3 for the seed 9 recruitment chain.

### Results

Overall, 1674 coupons were issued, including coupons for the 9 seeds, of which 1384 (82.7%) were returned within their valid period. Of the returned valid coupons, 98.1% were deemed eligible to participate and 100% of these participants consented to be in the study. Three participants (all self-administered) were excluded for excessive missing data (> = 20 items), (Table 1).

**Table 1:** Respondent Driven Sampling implementation parameters

Parameter	Number
a. Total # seeds	9
b. Total # coupons given	1665
c. Total # coupons returned outside of validation period (after expiration date)	36
d. Total # coupons returned within validation period (sum e, f, g)	1375
e. Of returned coupons, # ineligible total	27
# ineligible due to identified as attempted duplicate entry via fingerprint scanner	0
# ineligible for other reasons (age, marital status, residence)	27
f. Of returned coupons, # non-participating	0
g. Of returned coupons, # eligible and participating	1348
h. Total number of participants including seeds (sum a and g)	1357
i. Participants providing incomplete or nonsensical data	3
j. State the threshold and number % of individuals	Threshold of = > 20 items missed of ~ 100 survey items (0.2%)
k. Duplicate participants dropped	0
l. Lost or unsaved interviews	0
m. Final analytic sample (h minus I, k, and l)	1354
n. Number of recruits by seed, mean (range)	157.8 (4–245)
o. Number of recruitment waves, mean (range)	7.4 (2–10)

**Fig. 1:**
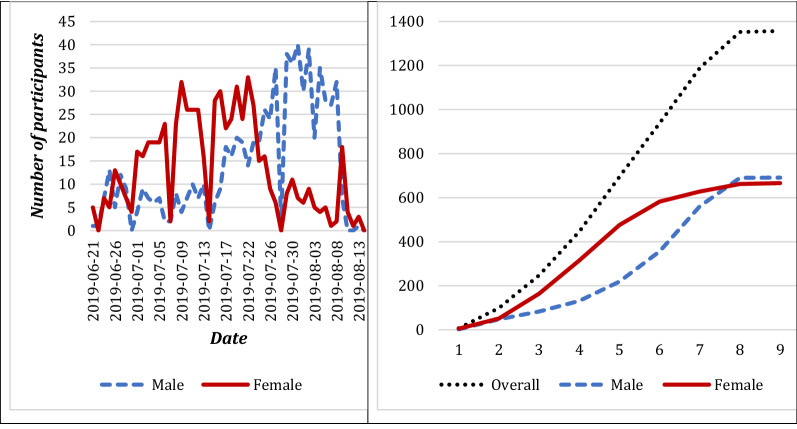
Daily and cumulative enrolment

**Fig. 2:**
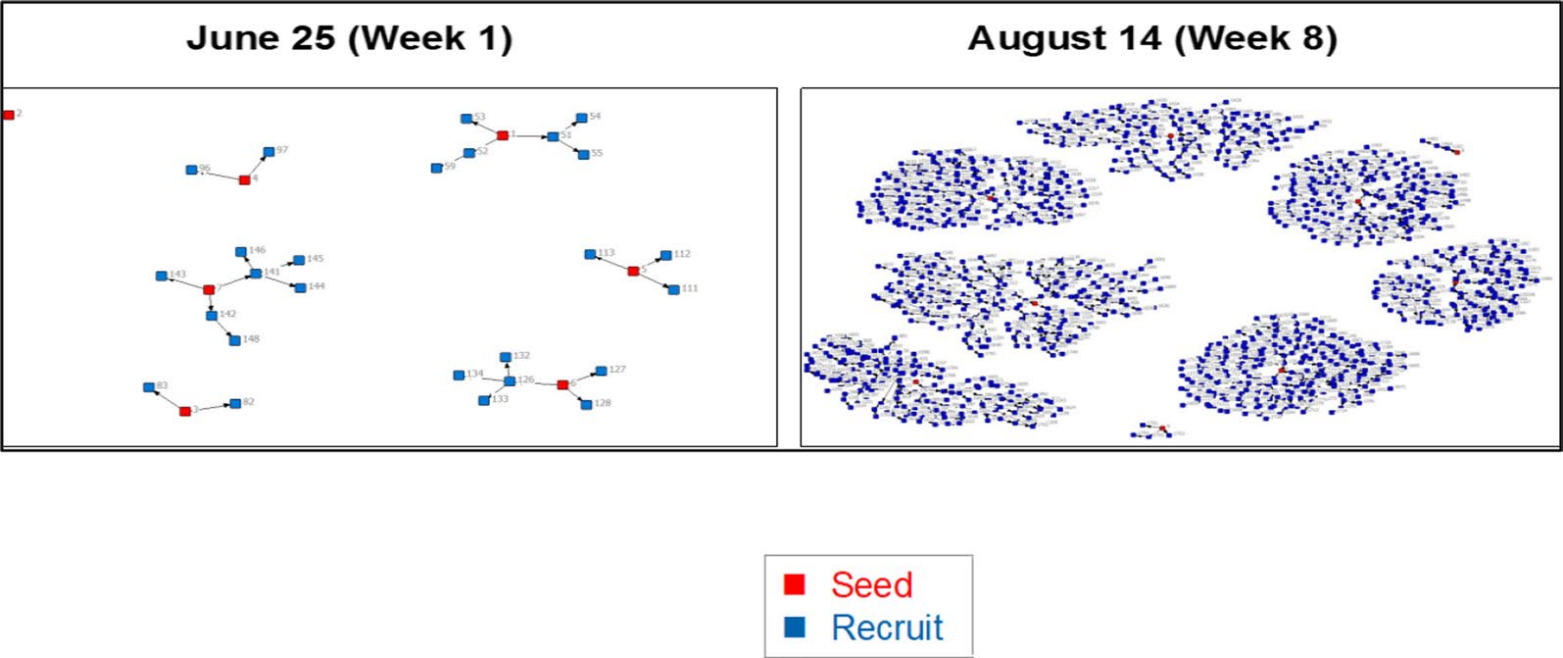
Recruitment chains

Figure 1 shows daily and cumulative enrolment by gender. This metrics was monitored closely to ensure enrolment remained on target. There was differential recruitment by gender in both pace and recruitment gender that resulted in more rapid female enrolment and necessitated the launch of 2 male booster seeds. About 56.0% of the female participants gave a coupon to a female recruit compared to 44.0% male participants who gave a coupon to a male recruit. As shown in Fig. 2, there were two very productive seeds and one unproductive seed in week 1, although by week 8, the seven initial seeds had generated long recruitment chains.

#### Data collection challenges

Recruiting seeds from affluent areas was challenging; the only one which was identified was not successful in growing their network. While study compensation was provided, it may not have been sufficient to engage youth from more affluent settings, and it is possible that competing priorities and comfort with data collection facilities may have also influenced the low participation in this segment.

Thirty-six individuals presented expired coupons, and five individuals attempted to enroll in the study but claimed they had misplaced coupons received. These individuals were not enrolled. Although eligibility information was provided on coupons, study participants distributed coupons to a number of ineligible individuals, including 27 who presented to the study team but were not enrolled in the study due to ineligibility (9 had stayed in Nairobi less than one year, 5 were not from Nairobi county, 5 were married/had a partner, 5 were over 24 years and 3 were below 15 years). The majority of participants were able to use the tablet (54.2%), following brief training (average 3 to 5 min).

#### The fingerprint scanner

Misuse of coupons and multiple participation was prevented by fingerprint scanning which identified who had already participated. Each day enrolments were synchronized for all study computers across the seven sites. There were no duplicate participants.

### Discussion

RDS was feasible and efficient in reaching adolescents and youth in a short period of time to achieve the desired sample size rapidly. Des Jarlais et al. [29] concluded that compared to snowballing, RDS was quite robust even with snowballing modification in reaching equilibria and low homophiles for major variables suggesting no bias. Mayo-Wilson et al. [30] study reported that RDS facilitated identification of underserved young adults who may have been missed by other sampling strategies.

There are key lessons learnt. First, as has been reported in RDS implementation with other populations, the study team must remain vigilant of individuals who will attempt to beat the system to access compensation given to study participants [11, 21, 26]. Though the amount for compensation was considered minimal, some may still consider it worth attempting to obtain; these individuals have been described as “hustlers and entrepreneurs” [11]. Training the research team to detect and respond firmly to attempted ineligible enrollments is essential. This must be done in a way that does not undermine trust with the underlying community nor the approachability of the study team. Use of the fingerprint scanner was effective in deterring attempts.

Second, recruitment must be monitored closely to ensure desired gender mix and sample size are achieved. Differential recruitment has been reported in other studies using RDS [11]. Overrepresentation of particular gender or segment of the target population could lead to biased results [11, 21]. To mitigate this challenge, researchers may want to consider gender stratification of coupons. In addition, too many valid coupons with potential participants can be a staffing and study nightmare [21]. The goodwill of the community may be lost, which may impact negatively the study results dissemination and implementation of the findings. Implementation of study findings require community support [31]. It is therefore important that coupon management is done in such a manner to ensure the study maintains traction with the community.

## Conclusions

We have demonstrated the feasibility and efficiency of using RDS to reach adolescent and youth. Despite the efficiency and feasibility of RDS for research with youth, several methodology features should be considered. The controlled recruitment, option to weight results, and analytic adjustment for non-independence of participants offer analytic strength and rigor, however these methods may not fully mitigate bias, such as overrepresentation of certain segments of the population. The efficiency of this method is likely most pronounced in urban settings. As alternate research methodologies evolve to capture youth voices as part of the broader attempts to address inequities and inequalities [32] in relation to SRH, RDS is one feasible option.

## Limitations

This study reports on RDS implementation parameters in urban Nairobi, Kenya. Results are likely most generalizable to large urban areas with high concentrations of unmarried youth and established relationships with the youth network that can support formative research as well as recruitment sites.

## Supplementary Information

**Additional file 1: **Performance Monitoring and Accountability Agile Youth Respondent-Driven Sample Survey (YRDSS**)**

## Data Availability

Youth respondent-driven sampling survey data are accessible on request through the project email address (kenya.agile.data@pma2020.org). However, data used for this analysis can be made available by the research team to researchers who meet the criteria for access to data.

## Abbreviations

*CAPI*: Computer Assisted Personal Interview
*PMA2020*: Performance, Monitoring and Accountability 2020
*RDS*: Respondent-Driven Sampling
*SRH*: Sexual and reproductive health
*YRDS*: Youth Respondent Driven Sampling
*FP*/*C*: Family Planning/Counselling
*MWRA*: Married Women of Reproductive Age
*UI*: Uncertainty interval
*LMIC*: Low-Income and Middle-Income Countries
