# Supplementary material for: Implementation of respondent driven sampling in Nairobi, Kenya, for tracking key family planning indicators among adolescents and youth: lessons learnt
Source: BMC Res Notes. 2022 Jun 7;15:200. doi: 10.1186/s13104-022-06038-8 (PMC9171948; doi:10.1186/s13104-022-06038-8)
Supplement: Supplementary file 1 — Additional file 1: Performance Monitoring and Accountability Agile Youth Respondent-Driven Sample Survey (YRDSS) [file 13104_2022_6038_MOESM1_ESM.pdf]

Performance Monitoring and Accountability Agile Youth Respondent-Driven Sample Survey (YRDSS)

Prof Peter Gichangi
Principal Investigator

Prof Amy Tsui
Co-Principal investigator

Scott Radloff
Co-Principal investigator

Mary Thiongo
Co-investigator

Table of Contents

Abbreviations 3

Introduction/background 4

Literature review 4

Aims of the Study: 6

Objective 6

Study specific objectives 6

Justification of the study 6

MATERIALS AND METHODS 7

Study setting 7

Study Design: 8

Study population 9

Study Procedures: 9

Survey instruments 11

Data collection 13

Data analysis 14

Ethical considerations 14

Consent Process: 15

Data Security and Confidentiality Protections: 16

Risks of the Study: 16

Direct Personal and Social Benefits: 17

Payment: 17

Training in human subjects research protections 18

Recordkeeping: 18

Safety Monitoring: 18

Reporting Unanticipated Problems/Adverse Events (AE's) to the IRB: 18

Significance of the study 18

Study limitations 19

Study Management: 19

Budget 21

Time lines 22

REFERENCES 23

Abbreviations

CPR	Contraceptive prevalence rate
DEFF	Design effect
ICRH	International Centre for Reproductive Health
ID	Identification
IRB	Institutional Research Board
JHSPH	John Hopkins School of Public Health
M&E	Monitoring and Evaluation
mCPR	Modern contraceptive prevalence rate
MOH	Ministry of Health
ODK	Open Data Kit
PI	Principal Investigator
PMA	Performance monitoring and accountability
RDS	Respondent Driven Sampling
YRDSS	Youth Respondent-Driven Sample Survey

Introduction/background

Respondent Driven Sampling (RDS) is a modified chain-referral recruitment sampling approach with a mathematical system for weighting the sample to compensate for its not having been drawn randomly. RDS begins its sampling strategy with a selected number of seeds. These seeds serve as the starting point for sampling. They recruit their peers based on the inclusion criteria for the study using a voucher system. The seeds and the peers they recruit complete the study survey, and then are asked to recruit up to three members of their network. Throughout data collection, and unlike other chain-referral approaches, RDS offers the means to evaluate the reliability of the data being obtained.

RDS is premised on the assumption that peers are better able than outreach workers and researchers to locate and recruit other members of a hidden population. Thus, RDS surveys have been widely used for hard-to-reach populations, including men who have sex with men, injection drug users, homeless youth and illegal immigrants and adolescents and youth in Abidjan, Kenya recently under PMA Agile (Badowski et al 2017; Gile and Handcock 2010). While typically indicated for hidden populations, RDS is similarly valuable for hidden *behaviors*. In settings where sexual activity and family planning for adolescents are intentionally hidden due to social and familial pressure, RDS can be a valuable means of recruiting adolescents for survey and intervention research on this hidden topic. In Nairobi, 17.5% of all unmarried females 15-24 in the recent PMA2020 household/population survey report currently using contraception, primarily emergency contraception (EC) and male condoms. PMA Agile typically monitors contraception uptake via clinic-based surveys and monitoring; however in this age population, it is suspected that young females and males may be procuring contraceptives via other means (e.g., via older friends), making young contraceptors effectively “hidden” to clinic staff, and compromising the accuracy of clinic-based survey methods. How young females and males procure their methods is not well known and it is assumed their sexual partners, relatives or other adults assist in procurement. Conducting a YRDSS will inform about youth awareness, use and acquisition of family planning methods among both female and male adolescents, and enable reach into a population and topic that may be otherwise hidden. This study will therefore be undertaken to describe awareness, usage and source of contraception among unmarried adolescents and youth in Nairobi.

Literature review

Adolescent pregnancy continues to be a major problem in Kenya, with a teenage pregnancy rate of 18 % and an adolescent birth rate of 96 per 1,000 women (KNBS, 2015). Data from the 2014 Kenya Demographic Health Survey (KDHS) indicates that one in every five teenage girls between the ages of 15-19 have begun childbearing; contraceptive prevalence rate among sexually active unmarried girls aged 15-19 years is 49%; the age of sexual debut has decreased, with 12% of young women and 21% of young men aged 15-24 years having had sexual intercourse before the age of 15, while 47% of young women and 55% of young men between the ages of 18-24 years have had sexual intercourse before the age of 18. The estimated number of girls (15-19) years old who began child bearing grew from 755,000 in 2009 to 843,000 in 2014, which is in part attributed to the high unmet need for contraceptives among adolescents, currently estimated at 23% (KNBS, 2015).

Adolescent pregnancy, whether intended or unintended, increases the risk of maternal mortality and morbidity, including complications of unsafe abortion, prolonged labor, delivery and post-natal period (Banke-Thomas et al 2017). In addition, consequences of unintended pregnancy among girls in Kenya include termination of education (dropping out of school), child marriage, and unsafe abortion (Rosemary and Martin, 2008). Evidence shows that among adolescent girls who had started childbearing by age 18 in Kenya, 98 percent were out of school (Rosemary & Martin, 2008). It is also estimated that about 13,000 girls drop out of school annually in Kenya due to early and unintended pregnancy (Rosemary and Martin, 2008). Women who become mothers in

their teens are more likely to curtail education and have reduced career progression and economic empowerment, hence perpetuating the cycle of poverty (KNBS, 2015).

Due to the social sensitivity surrounding ASRH issues, many countries lack formal policies regarding the provision of Reproductive Health (RH) information and services to adolescents. Kenya has developed a National Adolescent Sexual and Reproductive Health Policy (revised 2015), which provides guidance to government ministries and partners on how to respond to ASRH needs. This Policy recognizes the importance of addressing adolescent sexual and reproductive health needs for achieving the country's development goals. Despite this legal framework, implementation of ASRH services has been weak and uncoordinated (MOH, 2015). The absence of reinforcement of ASRH policies enables administrators and service providers to impose restrictions based on their personal beliefs that prohibit youth from gaining access to essential information and services. As such, contraceptive prevalence rate (CPR) among married adolescent girls (15-19) is low at 37% while the unmet need for FP among adolescents is high at 23% as compared with CPR of 56% among 15-49 with an unmet need for FP at 18% for the later (KNBS, 2015).

Adolescents' access to sexual and reproductive health services and information, is a gap that is yet to be fully addressed. Several studies have been undertaken to assess what works to increase access and utilization for ASRH among young people. There is however lack of scientifically sound data on the effectiveness of services targeting young people in Sub-Saharan Africa, in comparison to the magnitude of ASRH challenges experienced in the region (Denno et al 2015). In addition, there is limited evidence to support the effectiveness of initiatives that simply provide adolescent friendliness training for health workers (Denno et al., 2015). Results show that interventions with multiple components such as a combination of health service provider training, multi-sectoral collaboration, and community wide health education activities and outreaches services are often reported to lead to increased ASRH service use by young people (Denno *et al.,* 2015).

Systematic reviews of available published literature suggest that current interventions targeting youth tend to have significant positive effects on improving young people's knowledge and sometimes attitudes regarding sexual behavior, but are less effective in demonstrating change in sexual behavior outcomes (Chandra-Mouli et al., 2014). A review of Evidence on Interventions Commonly Accepted as Best Practices *What Does Not Work in Adolescent Sexual and Reproductive Health*, established that many ASRH interventions are implemented in an uncoordinated and piecemeal fashion, hence does not result in positive outcomes (Chandra-Mouli *et al.,* 2015). A study on young people's perception of ASRH services in Kenya showed that young people wished to see an increase in ASRH services, especially in rural areas, including the use of mobile clinics. The study also suggested the need to increase awareness of available ASRH services among young people and the community in general through outreach activities in the community, schools and churches (Godia *et al;* 2014)

However, there is very limited research that has been conducted to understand how boys and girls respond differently to existing RH needs and/or services. There is also limited research that has been undertaken with a focus on the adolescent boys. Some of the studies and programming limit their gender responsiveness only to data disaggregation by sex, but do not dig deeper to establish why boys and girls may respond differently to SRH services, and how/why the uptake of these services differs by gender.

The 2015 ASRH policy for Kenya advocates for working with ministries of Education and Health among other line ministries, inclusion and participation of young people, political arms and others stakeholders for the success of ASRH programs (Kenya, 2015)). To actualize the 2015 policy, the Ministry of Health outlined four service delivery models for ASRH in Kenya which include: a) Community based: Services and information are

offered to adolescents within the community/non-medical settings b) Clinical based: Services and information are offered to adolescent within/based on health facility setting c) School based: Services and information are offered to adolescents within the school setting. d) Virtual based: Services and information are offered to adolescents within the virtual space or digital platforms. Implementation of this combined model has not been fully implemented, and its effectiveness has not yet been established in Kenya.

There is need to determine awareness, usage and source of contraception among unmarried aged 15-24 years in Nairobi county in the context of all interventions being implemented in Nairobi County which seeks to improve access and utilization of quality ASRH services and consequently reduce the burden of teen pregnancy.

Aims of the Study:

The Performance Monitoring and Accountability 2020 (PMA2020) Project is being implemented by the Bill & Melinda Gates Institute for Population and Reproductive Health at the Johns Hopkins Bloomberg School of Public Health. PMA2020 supports regular low-cost, rapid turnaround, nationally-representative surveys using mobile technology to gather, analyze and disseminate health information at both household and facility levels. PMA Agile is a separate but related three-year grant that has been developed to capitalize on PMA2020 to build a monitoring and evaluation platform for large-scale projects that will enable near-continuous tracking of family planning performance and progress toward their intended results. PMA Agile tracks change at the health system level through quarterly public and private health facility audits and periodically through the conduct of client exit interviews about contraceptive behaviors. PMA Agile is operational in seven countries in Africa and Asia, including Kenya, working through local university and research organizations with the aim of building local capacity.

PMA Agile seeks a reliable means for monitoring family planning awareness and use among youth as they enter a period of probable sexual activity and capturing this information from youth clients of health facilities, especially unmarried females, is challenging due to social and familial pressure to hide sexual activity and family planning. Thus, the project aims to conduct a Youth Respondent-Driven Sample Survey (YRDSS) in collaboration with a youth-serving non-governmental family planning organization in Nairobi, Kenya, following the successful implementation of a pilot YRDSS in Abidjan, Côte d'Ivoire in August-November 2018 (JHSPH IRB no: **IRB00008863**).

Objective

To determine awareness, usage and source of contraception among unmarried aged 15-24 years in Nairobi county.

Study specific objectives

The overarching specific objectives of the study are as follows:

1. To estimate the percent of unmarried females and males aged 15-24 years aware of different methods of family planning
2. To estimate the percent of unmarried females and males aged 15-24 years using family planning methods
3. To understand the sources of and consumption patterns of family planning methods among unmarried females and males aged 15 to 24 years

Justification of the study

Respondent-driven sampling (RDS) is a modified chain-referral recruitment sampling approach with a mathematical system for weighting the sample to compensate for its not having been drawn randomly. RDS

begins its sampling strategy with a selected number of seeds. These seeds serve as the starting point for sampling. They recruit their peers based on the inclusion criteria for the study using a voucher system. The seeds and the peers they recruit complete the study survey, and then are asked to recruit up to three members of their network. Throughout data collection, and unlike other chain-referral approaches, RDS offers the means to evaluate the reliability of the data being obtained.

RDS is premised on the assumption that peers are better able than outreach workers and researchers to locate and recruit other members of a hidden population. Thus, RDS surveys have been widely used for hard-to-reach populations, including men who have sex with men, injection drug users, homeless youth and illegal immigrants. While typically indicated for hidden populations, RDS is similarly valuable for hidden *behaviors*. In settings where sexual activity and family planning for adolescents are intentionally hidden due to social and familial pressure, RDS can be a valuable means of recruiting adolescents for survey and intervention research on this hidden topic. PMA Agile typically monitors contraception uptake via clinic-based surveys and monitoring; however in this age population, it is suspected that young females and males may be procuring contraceptives via other means (e.g., via older friends), making young contraceptors effectively "hidden" to clinic staff, and compromising the accuracy of clinic-based survey methods. How young females and males procure their methods is not well known and it is assumed their sexual partners, relatives or other adults assist in procurement. Conducting a YRDSS will inform about youth awareness, use and acquisition of family planning methods among both female and male adolescents, and enable reach into a population and topic that may be otherwise hidden.

MATERIALS AND METHODS

Study setting

Nairobi County is one of the 47 counties in the Republic of Kenya. It borders Kiambu County to the North and West, Kajiado to the South and Machakos to the East. The County has a total area of 696.1 Km2 and is located between longitudes 36o 45' East and latitudes 1o 18' South. It lies at an altitude of 1,798 metres above sea level.

In 2009, the County population was projected to be 3,138,369 and is expected to rise to 4,941708 in 2018, 5,433,002 in 2020 and 5,958,338 in 2022 respectively, table 1. The table further indicates that the female population projections from age cohorts 0-4, 5-9, 10- 14, 15-19 and 20-24 remain slightly higher than that of male except for under 5 where the number of boys is higher than that of girls. From the age bracket 35-39 the population of males overtakes that of females and remains higher up to the age bracket 75-79.This is attributed to influx of men from rural areas to Nairobi in search of white colour jobs. Above 80+ years the female population remains higher than the male counterparts over the years stipulated in the table. This is a result of life expectancy where men's life expectancy is shorter than women. Further research should be done to find out the factors which lead to this phenomenon. The population distribution shows a pyramid that is heavy at the base, with the population less than 15 years being approximately 9 per cent in 2020 and 80+ being 0.2 percent.

Table 1. Nairobi population projections

Age Cohort in yrs	2009 (Census)			2018			2020			2022		
	Male	Female	Total	Male	Female	Total	Male	Female	Total	Male	Female	Total
0-4	199,381	197,780	397,161	365,338	370,333	735,671	380,748	377,194	757,942	412,538	418,359	830,897
5-9	151,900	154,977	306,877	255,744	258,356	514,100	333,509	335,141	668,650	361,355	371,717	733,071
10-14	119,951	127,014	246,965	192,295	219,110	411,405	239,847	248,647	488,494	259,873	275,783	535,656
15-19	115,772	154,292	270,064	163,550	228,291	391,841	202,204	252,562	454,766	219,087	280,125	499,212
20-24	211,089	266,307	477,396	225,060	288,963	514,023	205,308	282,048	487,356	222,450	312,829	535,279
25-29	234,596	228,157	462,753	311,342	367,292	678,634	239,306	296,112	535,418	259,286	328,428	587,715
30-34	182,623	141,506	324,129	265,408	296,256	561,665	277,023	326,634	603,657	300,153	362,281	662,434
35-39	134,459	95,173	229,632	204,494	173,026	377,520	228,376	255,529	483,905	247,444	283,416	530,860
40-44	89,109	57,492	146,601	158,326	116,851	275,176	173,861	148,954	322,815	188,377	165,210	353,587
45-49	65,901	41,102	107,003	102,893	73,123	176,017	133,206	100,228	233,434	144,328	111,166	255,494
50-54	41,682	24,894	66,576	70,471	47,848	118,320	85,780	63,199	148,979	92,942	70,096	163,038
55-59	24,304	14,981	39,285	44,151	30,811	74,962	57,781	41,616	99,397	62,605	46,158	108,763
60-64	15,061	10,105	25,166	27,617	20,138	47,755	35,555	27,106	62,661	38,524	30,064	68,588
65-69	7,358	5,664	13,022	15,259	12,866	28,125	21,761	17,700	39,461	23,578	19,632	43,210
70-74	4,462	4,240	8,702	8,409	8,105	16,514	11,562	10,994	22,556	12,527	12,194	24,721
75-79	2,424	2,519	4,943	4,144	4,908	9,052	5,889	6,502	12,391	6,381	7,212	13,592

Source: Nairobi County Integrated Development Plan 2018-2022

Study Design:

This will be a cross-section study design using Respondent-Driven-Sampling (RDS), which is similar to snowballing, but corrects for the bias through statistical adjustments (Badowski et al 2017). Respondent-driven sampling begins with the selection of "seeds" i.e. known members of the population of interest. Each seed will asked to identify a number of other AYWs from their social network for the survey, who subsequently identify other AYWs, and son on in waves. Each wave of participants subsequently recruits the next wave of participants until the recruitment goal is reached (Johnston et al., 2016).

An initial sample of 20 seeds will be used to begin the YRDSS. Seeds are typically chosen by referral from key informants during the formative research phase on the basis of their meeting inclusion criteria established for

the study and demonstrating a willingness to recruit their peers for the study. These seeds and the peers they recruit will be asked to present at the local project office where their informed consent will be reviewed and processed, and they will complete an anonymous, self-administered survey. They will then be asked to recruit up to three members of their network using the coupons. Participants are invited to check back to the project office for a secondary incentive for each successful recruit.

Study population

This study will be done in Kenya, Nairobi county and will involve adolescents and young women aged 15-24 years who are not married. Languages to be used wil be Englsih or Swahili.

Sample size

We will begin with approximately 20 seeds. The target sample size for this pilot is estimated to be 1300 females and males ages 15 to 24 years. This is based on the observed modern contraceptive prevalence rate of 17.4% among unmarried females 15 to 24 years of age in PMA2020/Nairobi, 2017. The simple random sample size required for a +/- margin of error of 3% points is 614. The design effect (DEFF) is 2, which results in an effective sample size of 1228. With a 5% field recruitment error rate, the target sample size is 1293, which we have rounded to 1300.

Participants:

The YRDSS sample size is estimated to be 1300 unmarried females and males ages 15 to 24 years, who will be recruited through selected seeds, approximately 20 in number. In accordance with best practices for RDS, seeds are members of the target population and represent diversity with regard to underlying subpopulations. The on-site planning and training phase will be used to refine characteristics for purposive seed selection; these are anticipated to include age range (e.g., 15-18; 19-24), gender, education, and geography within the study site.

All seeds and recruited participants will be residents of Nairobi and will give consent to participate.

Inclusion criteria

1. Unmarried male or female, aged between 15-24 years old
2. Lives within the geographic boundaries of the city (Nairobi, Kenya)
3. Able to present a valid recruitment coupon

Exclusion criteria

1. Male or female, not between the ages of 15-24 years old or married
2. Does not live within the geographic boundaries of the city (Nairobi, Kenya)
3. Unable to present a valid recruitment coupon

Study Procedures:

No JHSPH staff members will be directly involved with data collection. Rather, JHSPH will provide overall technical assistance on tool development, study implementation, and data management. ICRHK and the in-country team supervise fieldwork during the data collection period.

<u>Recruitment Process</u>:

This study will recruit using RDS. RDS provides a means of selecting a sample and also allows interpretations about the characteristics of the population from which the sample is drawn. The ability to achieve the broad coverage offered by RDS is dependent on sampling individuals whose membership in the group of interest creates connections among the larger population. RDS has demonstrated the ability over the years to effectively and efficiently recruit among marginalized or hard to reach populations. To mitigate against unknown barriers to participation and recruitment (e.g., geographic, cultural, demographic), seeds will be recruited in varying geographic locations throughout Nairobi, varying gender and age groups, with varying social subgroups and networks.

Recruitment will begin with a limited number of seeds who will be identified by key informants or project staff. ICRHK works closely with organizations that supports youth groups in Nairobi County and is part of a network and technical working group of local organizations that conduct youth outreach to different populations of youth in Nairobi. The youth groups and community based youth-serving organizations can help generate seeds and serve as key informants for additional seeds.

All seeds will be well-connected socially with other adolescents. In addition, seeds will be diverse with respect to demographic and geographic factors. In accordance with RDS best practices, seeds can be added as needed throughout the study. If an important social network from which no seeds were drawn is discovered during the course of data collection, and if the ongoing recruitment does not eventually include significant members of that group, seeds can be added from this group. Seeds and subsequent recruitment networks will be monitored for propagation (i.e., ongoing participant recruitment); additional seeds may be added following the methods described for recruitment chains that die out (i.e., that do not continue to generate participants).

Using the eligibility criteria previously described, project staff will recruit an initial group of seeds from various locations within Nairobi. Research staff will briefly present the study and eligibility outreach staff at ICRHK and other local youth-serving organizations who will serve as key informants to identify dynamic youth eligible for the study and refer them to research staff for more information. When a potential seed is identified or contacted, research staff will briefly describe the project and ask the potential seed if he/she would be willing to learn more about the project. Research staff will then have an informal discussion with the potential seed to determine if this individual meets the eligibility criteria as well as the characteristics previously described. If the individual agrees, they will complete the informed consent process and complete all study procedures at the hotspot nearest to them, at a time deemed to be mutually agreeable.

Once a seed consents and completes the survey questionnaire, a brief 1-2 minute, individual training on the recruitment process will be conducted. Each seed will be given up to three coded, non-replicable coupons. This method has been used successfully in past RDS research. The respondent will be invited to give one coupon to each of 3 unmarried adolescents between the ages of 15 and 24 with whom he/she is connected who fit the previously described eligibility criteria. Coupons will list phone numbers for the 13 resident enumerators, the address of their hotspot locations throughout the city, and their study hours.

A few safeguards will be put in place to prevent duplicate enrollments. Crosschecking ID cards against consent forms will provide confirmation on participant identity and will also serve as a deterrent to dual enrollment. If a recruit does not have an ID card on them at the time of enrollment, a short checklist will be used to verify the participant's age. Careful selection and training of the coupon manager and the use of continuous staff will also be employed to avoid opportunity for attempted repeats.

Privacy issues related to recruitment
There are minimal privacy issues associated with recruitment. All participants who opt to receive their incentives through M-Pesa will need to provide a phone number to facilitate the money transfer. The phone number provided will only be used for this purpose. Participants will have the option to provide their first name and phone contact if they wish to participate in the follow-up study. Providing contact information for the follow-up study is optional. If the participant opts to not provide information for the follow-up study, the phone number used to transfer their incentive(s) will be destroyed following the study completion. Seeds and their peer recruits will be given unique participant IDs that will be associated with their coupon vouchers.

When discussing potentially sensitive questions related to adolescents' use and awareness of family planning methods, and relationship dynamics, youths will be reminded that they do not have to answer any questions they do not feel comfortable answering. They will also be reminded that all answers will remain anonymous and kept confidential. If a participant presents to a study hotspot to receive secondary incentives for successfully recruited participants, they will not be provided with information on which of their referred participants completed the study.

Survey instruments
The questionnaires are prepared in Open Data Kit (ODK) software for downloading and use on Android tablets. After having consented to participate, all eligible participants will complete the self-administered survey. The questions will have separate wording for females and males as necessary. The instrument collects information on various reproductive health issues, including knowledge, attitudes, use and acquisition of contraception. The survey should take approximately 30 minutes to complete. Participants have the option to refuse to answer any question they do not wish to answer. Once completed, the participant will close the survey, which will be stored on the tablet for transmission by a project associate to a secure cloud server. Should the participant have difficulty navigating the survey questions on the tablet, a staff interviewer will be available to assist with questions as well as completion of the survey, i.e., should the participant wish to revert to having the interviewer ask remaining questions. Survey instruments and consent forms will be available in English and Swahili languages.

The survey instrument will be pretested prior to use in order to maximize comprehension by the study participants. Logic checks and skip patterns will be built into the ODK form to minimize errors in data capture. Data checks will be done on a weekly basis to ensure the desired coverage of items during the data collection period. Project meetings between in-country and Baltimore team members will also be held in a timely manner to identify any problems the participants faced while responding to the survey or the study team encountered with data processing.

Brief (1-2 minute) training on peer recruitment
Following completion of the survey, each participant will be provided with up to three coupon vouchers. Resident enumerators will ask participants to think of (but not name) at least three of their peers who meet the eligibility criteria for the study, and whom they can imagine inviting to participate. The youths will be given a brief (1-2 minute), individual training on how to present the study to their peers and invite their participation. Coupons handed out for distribution will be linked with participants through ID numbers only, to preserve anonymity while enabling the tracking of recruitment networks. The coupon stub provided to participants enables tracking of this recruitment chain process.

Primary and secondary incentives

All participants who successfully complete the survey will receive a primary incentive of $5 to compensate them for their time and participation. Participants who successfully recruit peers into the study within the specified time period via study procedures are eligible for a "secondary" incentive of up to $9, i.e., reimbursement for successfully recruiting up to three additional participants. After the window of time in which participants can distribute their coupons has passed, participants will receive the secondary incentive for each eligible participant recruited.

Both primary and secondary incentives will be distributed using M-Pesa, a mobile phone-based money transfer system, which was decided based on the study partner's strong recommendation due to its widespread and accepted use among youth in Nairobi. To receive incentives via M-Pesa, participants will need to provide the phone number that is connected to their M-Pesa account to the study staff. The use of the phone number as a link with the M-Pesa account enables our study team to safely and efficiently transfer funds to participants without obtaining additional identifiers such as bank account numbers. M-Pesa is widely used in Nairobi; this system reduces the risk to participants and staff of traveling with cash. The phone number will be linked to their coupon ID number to maintain confidentiality. At the end of a study day, all M-Pesa transactions for primary incentives will be submitted at the same time by a member of the central study team. Secondary incentives distributed via M-Pesa will be similarly submitted to all eligible participants at the end of a specified weekday by a member of the central study team. If a participant does not have an M-Pesa account, they will have the option to receive their incentives in the form of cash at the hotspot. Their ticket stub provides information to contact study team to check on the status of their secondary incentives if needed.

STUDY IMPLEMENTATION:

After providing consent to participate, all eligible participants will complete the self-administered survey questionnaire. The instrument will collect information on reproductive health topics, including knowledge, attitudes, relationship dynamics, use and acquisition of contraception as well as demographic characteristics. The survey should take approximately 30 minutes to complete. Participants will have the option to refuse to answer any question they do not wish to complete.

After completion of the survey, the participant will be invited to recruit other participants. Those who agree will be given a brief 1-2 minute training on the recruitment process, and then will be given up to three coded, non-replicable coupons with an expiration date (generally within 2 weeks of participation). The respondent will be instructed to give one coupon to each of up to three unmarried youth between the ages of 15 and 24 within the community. The participant will also receive a coupon "stub" to keep that enumerates the participant's own ID number, the numbers of the three coupons s/he was given, and their expiration date, in addition to a brief coupon referral script that contains the same information about the recruitment process that was shared with them in-person by the study staff.

If the participant opts to receive their secondary incentive via M-Pesa, the participant does not need to return to the study hotspot for their secondary incentive. Study staff will track how many of the participant's recruits participated in the study during the coupon eligibility window and transfer the secondary incentive following the end of the expiration period. If the participant does not use M-Pesa to obtain their secondary incentive, the participant will be instructed to return to the same study site during hours designated on the coupon stub after the coupon expiration date to inquire about and receive secondary incentives for any recruited participants. This process of recruitment and interviewing will continue until the RDS target sample size of 1300 adolescents is achieved. A resource referral sheet with information about family planning, other reproductive health and

social welfare services information will be provided to all participants. Finally, participants will be invited to provide contact information for themselves and two additional individuals knowledgeable about how to reach them to participate in the 6-month follow-up survey, at which point they will be asked again to consent to participate in this survey. We are in the process of securing funds for this follow-up phase.

Note: We specify "up to three" coupons per participant; most participants will receive three coupons to distribute however in the final stages of recruitment, number of coupons provided will taper to 2 then 1 then 0 in order to wind down the recruitment period.

The total number of participant visits to the project office should exceed no more than two. The first visit includes informed consent, survey completion and training on participant recruitment, with the entire visit lasting no more than one hour. Participants are invited to recruit other youth participants and receive up to three coupons to distribute. For each recruited participant (up to three), participants are eligible for a secondary incentive. We anticipate that most participants will opt to receive their primary and secondary incentives via M-Pesa, which eliminates the need for a second visit to the study site. For participants opting to receive the secondary incentive(s) in cash, participants may return to the study location and present their coupon stub with their study ID and coupon numbers, with the entire visit taking approximately 15 minutes.

Youths consenting to participate in the study will be seen no more than twice over an expected data collection period of 1 month. The duration will depend on the pace of recruitment. The expected overall duration of the entire YRDSS pilot is 6 months. This includes the planning phase, data collection and analysis.

RDS network recruitment patterns will be monitored on an ongoing basis to identify needs for additional seeds and overall data quality. PMA Agile/YRDSS will be responsible for cleaning the data and calculating survey weights, evaluating and selecting the appropriate RDS estimator, computing frequencies, proportions, and means or medians. Variables include the proportions of 15-24 unmarried females and males aware of and using different methods of family planning and the proportion of 15-24 unmarried females and males purchasing contraceptive methods themselves, as well as the sources they access.

While the main analytic purpose of the survey is to obtain aforementioned family planning indicators on unmarried female and male youth ages 15 to 24 in Nairobi for monitoring, there will be additional analyses conducted for the purpose of exploring avenues of focus for resulting interventions as well as factors that influence the key outcomes. Once weighted, the youth data will be analyzed for correlates of sexual experience, contraceptive method knowledge, use experience, and procurement behaviors. Correlates of interest are gender, age, partnership status, relationship dynamics, schooling, and social network influences. A topic of primary interest is how these covariates are associated with the facility source of contraception, type of purchaser (e.g., self, partner, adult relative) and cost. Multivariate regression models will be fitted and estimated for these analyses.

Outside parties interested in secondary data analysis can be added to the study investigator list in the future once eligible or provided with de-identified data.

Data collection

Data collection procedures included eligibility screening, obtaining informed consent from participants, and a detailed self administered interview. A paper-based coupon management system was used in the RDS sampling events to record information on the redeemed and distributed coupons and the receipt of primary and

secondary incentives. The recorded coupon tracking information was entered into Microsoft Excel after each event in order to track the recruiter-recruit relationships and the number of waves completed for each seed.

The interviews started with a series of questions that measured characteristics of participants' social networks among the target adolescents and young women. These data were collected in order to conduct RDS analysis and to assess how well the RDS assumptions were met in the study. Network sizes were measured by responses to the first question that asked, To further assess the RDS assumption of reciprocal relationships, participants (excluding seeds) were asked again about their relationship to the person who gave them the coupon (e.g., friend or acquaintance, co-worker, etc.) and whether or not they would have considered referring the person who referred them.

Data analysis

We will use the Respondent Driven Sampling Analysis Tool (RDSAT) version 7.1 (Volz et al., 2012) to calculate sample proportions, estimate population proportions and 95% confidence intervals, and average network sizes (i.e., dual component network size). The RDSAT option regarding the number of re-samples for bootstrap will be set to 1,600, and the pull-in outlier option will be used to minimize the effect of outliers in social network sizes (upper and lower 3%). The default will be used for all other RDSAT options. We will also examine homophily and equilibrium on key variables in RDSAT to assess the effectiveness of RDS in recruiting AYW. Homophily estimates measure the likelihood that recruiters recruit individuals with a similar characteristic to themselves rather than at random (Montealegre et al., 2013). Homophily values range from −1 to 1, with 1 indicating that participants from a particular subgroup recruited entirely within that subgroup and negative values indicating avoidance for recruiting individuals from the same subgroup. Equilibrium is the point at which the proportion for a sample characteristic remains stable despite additional waves of recruitment. An equilibrium distribution indicates that the resulting sample is independent of the initial non-randomly selected seeds and starts to represent the characteristics of the population (Heckathorn, 1997; Townsend et al., 2010). In this study, equilibrium will be considered reached when the change in sample proportions between waves was less than 2%, which will be the RDSAT default. The key variables we will selecte to generate population estimates and assess homophily and equilibrium will included sociodemographic characteristics (e.g., sex, age, etc), as well as variables relating to awareness, use and sources of contraception. Variables on participants' network characteristics (e.g., relationship to the recruiter), lifestyle variables, and some supplemental data regarding awreness, use and source of contraception will be analyzed using SAS (SAS Institute Inc., Version 9.4, Cary, North Carolina, USA).

Ethical considerations

There are minimal privacy issues associated with recruitment. Potential seeds will have the option to provide their first name and phone contact for ease of logistics in communicating with the research staff; any names and phone numbers will be destroyed at the time of data collection. Seeds and their peer recruits will be given unique participant IDs that will be associated with their coupon vouchers. When discussing potentially sensitive questions related to adolescents' use and awareness of family planning methods, and relationship dynamics, youths will be reminded that they do not have to answer any questions they do not feel comfortable answering. They will also be reminded that all answers will remain anonymous and kept confidential. When participants present to the study office to receive secondary incentives for successfully recruited participants, they will not be provided with information on which of their referred participants completed the study.

Consent Process:

The interviewer or another member of the study staff at the hotspot will conduct the informed consent process. All study staff will be well trained on the study protocol and procedures and will have received human subjects research and ethics training.

First, all potential participants must provide a valid recruitment coupon; seeds must provide a valid staff referral card or be accompanied by the project staff who recruited them (seeds only). The project staff will check the coupon to ensure it has not been voided or used previously and will check the date on the coupon to ensure that it has not expired. Second, project staff will conduct a brief eligibility assessment. After eligibility is determined, study staff will obtain informed consent from participants by reading the consent form, answering any questions, and obtaining their signed consent to participate. The consent discussion will take place in a private space within the hotspot as a one-on-one discussion between the participant and a member of the study staff. Participants will have the opportunity to have questions answered prior to completing the consent process. Study staff will address any questions that participants may have, prior to starting the survey.

Eligibility for study participation is assessed by first confirming the validity of the recruitment coupon, then confirming the participant's age via identification card, and oral confirmation that the participant is not married and resides in Nairobi. Seeds will be instructed to tell their recruits to bring a form of identification with them to the study site; this information will be reinforced by coupon managers when participants receive their outgoing coupons to distribute. If a participant does not have an ID card on them at the time of enrollment, a short checklist will be used to verify age eligibility.

The participant will sign the consent form unless the participant is unable to. If that is the case, oral consent will be obtained, the participant's thumbprint will be applied to the consent form, and a witness to the consent will sign the consent form.

Parental consent and assent from minors ages 15-17 was waived in the YRDSS pilot study in Abidjan by the JHSPH IRB (IRB00008863) on suggestion of the JHU IRB. Similarly, in this study, we request a waiver of parental consent for youth aged 15 to 17 pending review and approval by the Kenyan IRB.

In Kenya, 18 years old is a legal age of consent for the purposes of research, but a waiver of parental consent is requested as the proposed research has minimal risk to participating adolescents. Circumstances of stigma or shame for participants, due to the sensitive nature of the topics are of concern, therefore, we are requesting a waiver of parental consent, which means the adolescent boys and young men themselves will be asked to provide informed consent. A similar research with adolescent boys and young men was done in Abidjan and received JHSPH IRB approval - (IRB00008863) (using the same methodologies). Requested for waiver of parental consent is informed by our concern that the youth will feel inhibited about sharing the vulnerability they feel to engaging in risky behaviors if their parents have been consented (and hence might ask them to share research information or questions). Given the anonymous nature of much of the data collection (e.g. self administered questionnaire) and the minimal invasiveness of the proposed topics and methods, we are requesting waiver of parental consent.

We request that parental consent be waived in this study for youth ages 15-17, as well, pending review by the JHU IRB. We request this waiver for feasibility considerations and given that a category of youth ages 15-17, living apart from their parents or guardians and managing their own financial affairs, are considered mature

minors. Under a waiver of parental consent, participants who are minors (15-17 years) would undergo the same consent process as participants aged 18-24. After eligibility is determined and confirmed, study staff will obtain informed consent from the youth by reading the consent form and obtain their signed consent to participate.

Data Security and Confidentiality Protections:

YRDSS will follow approved PMA2020 and PMA Agile data security measures. Data collection will be done using tablets programmed with logic rules and skip patterns for quality assurance. All tablets will be encrypted and have a secure log in. Additionally, all applications that are used for data collection, including the internal storage, file management system and ODK require a separate password to open and access the app.

Upon completion of the interview, data will be sent to and stored on a secure, password-protected server, ODK Aggregate within the Google Cloud Platform. The server instances are managed by the research team at JHU and only individuals authorized by the PI will be able to access the data. During data transmission, all data are encrypted and the identity of the server is verified through the SSL certificate. ODK Aggregate does not have known security vulnerabilities and is actively maintained by IT professionals. A daily report with number of surveys conducted against expected will be generated. If the data are shared with outside investigators, all verifiable IDs will be deleted.

These data are not intended for research purposes, but as noted will be made publicly available for secondary analysis. Only de-identified data will be released in such cases. In order for data to be shared, a formal request will be submitted to the JHU/PMA Agile team and approved by the Study PI. A Data Use Agreement will be signed by the sharing and using parties. The Data Use Agreement will require investigators to agree to honor the terms and limits outlined in the original consents, agree to not try to re-identify study participants, and agree to not sell or share data with others. The investigators who request the data will be required to obtain the appropriate IRB approvals for secondary data analysis.

The analytic dataset will not contain any identifiers. The only PII retained in this study will be name and signature on the participant consent forms, which will be stored in a locked cabinet at the ICRHK main office. We anticipate that most participants will provide phone number for the purpose of receiving their primary and secondary incentives via M-Pesa; phone numbers linked with study ID numbers will be retained until the study closes baseline recruitment and all primary and secondary incentives are paid out; we will allow one additional month following the close of baseline recruitment to allow us to respond to any participant with questions about their secondary incentives. One month after close of baseline, phone numbers in the incentive database will be destroyed. The final piece of PII retained is the contact information and study ID for participants who opt to participate in the follow-up data collection at 6 months.

Risks of the Study:

<u>Youth survey:</u> There is a small risk of psychological distress since some questions may be viewed as being personal or sensitive, such as questions about family planning practices and relationship dynamics. The questionnaire takes approximately 30 minutes to complete. The client is assured that participation is voluntary and that he or she may end the survey at any time. The participant is informed that while every effort is made to protect the confidentiality of their answers, there is no absolute guarantee secure data will not be breached. We will also remind the participant that based on their network recruitment, members of their network may learn of their participation in the survey.

We anticipate the frequency and severity of potential harms described above as relatively minimal.

All participant surveys are self-administered. Interview staff will also be available on site to aid participants through any concerns. All interviewer staff will receive training in the ethics of human subjects research, including privacy and confidentiality of data, as well as the conduct of sensitive data collection with the target population. We provide a resource referral sheet to all participants to make them aware of support services available.

Interviews typically take about 30 minutes to complete and are conducted at different hotspots throughout the city. The primary burden associated with this study is time; however, to minimize this burden, activities will be held at multiple (13) locations throughout Nairobi that should allow recruits from different areas of the city to visit a site that is most conveniently located for them. Incentives and travel reimbursement will be offered to participants.

Surveys will be self-administered except when the participant requests assistance from a staff interviewer. When prompted to answer potentially sensitive questions, youth will be reminded that they do not have to answer any questions they do not feel comfortable discussing. They will also be reminded that there will not be any negative consequences for choosing to skip a question or topic area. If the questionnaire needs to be administered by a study staff member, survey administration will take place in a private location.

Direct Personal and Social Benefits:

There are no direct benefits to respondents for participation in YRDSS surveys but some youth may feel participation is a form of public service to provide information toward improved family planning service access, especially for their peers.

The information provided by participants will be used to improve knowledge of the factors that influence family planning awareness, use and access among unmarried youths (15 to 24) in Nairobi. This project will also promote the RDS approach for future monitoring and evaluation of youth family planning and reproductive health programs.

Payment:

Payment in the form of a money transfer via M-Pesa or cash, with the equivalent value of 5 USD will be given to each participant during the initial visit to complete the survey. A secondary incentive with the equivalent value of 3 USD will be given to each respondent for every successful recruit who completes the survey, for a maximum value of 9 USD. The study will separately reimburse for travel because participants must travel to the local partnering organization to complete the survey. A total of 5 USD will be reimbursed to each participant for his/her travel for each visit.

Remuneration expenses are expected to total an approximate maximum amount of $19 per respondent--$5 for the primary and up to $9 for the up to 3 secondary incentives, plus $5 for travel reimbursement for the first trip. We estimate that only 1/10 of participants will not have an M-Pesa account and will need $5 travel reimbursement for their second trip. Participants are able to terminate the interviews at any time and thus, there are no adverse consequences if the surveys are not completed. The full incentive of $5 and travel reimbursement will be provided irrespective of partial completion of the questionnaire.

Training in human subjects research protections

All investigators and anyone with access to respondents or their identifiable information will complete CITI training or the equivalent for their organization. The local co-investigator and field staff will be trained in human subjects research protections, by one or more members of core team. The training will follow JHSPH Ethics Field Training Guide and conform to the in-country requirements.

The research team will have regular engagement through regular phone, Skype calls, and email exchanges. The lead investigator will have the overall responsibility of implementing the study with the support of co-investigators. Program Management staff will assist with administrative and technical assistance throughout implementation, including coupon management, tracking of seed selection and movement, survey instruments, handling payments, and equipment maintenance.

Recordkeeping:

Fieldwork teams will receive training in ethics of research, data collection, storage, and confidentiality. Informed consent will be obtained from all study participants prior to administering the survey. All data are captured electronically. At the close of data collection, the memory of the tablets will be wiped. Staff are asked to ensure that every completed interview is immediately submitted to the cloud server. IRB will be informed of any protocol deviation should they occur.

Safety Monitoring:

There is limited contact time with the respondents since the questionnaires are relatively short. Youths are invited back after the data collection period (~ 4 weeks maximum) to collect their second payment for each successful recruitment. During the consent process, all respondents are provided with a study PI name and telephone contact information, whom they can call if any issues related to their participation arise. The local staff in country are responsible for reporting any adverse events to the IRBs. There is no medical monitor required for this study.

Reporting Unanticipated Problems/Adverse Events (AE's) to the IRB:

The informed consent form include the name and phone number of the in-country PI. The in-country PI is responsible for reporting adverse events to the local RB on record and to the JHSPH PI within three days of occurrence.

Significance of the study

Young females and males are susopected to access and use contraceptives from non-clinic fcailities i.e. via other means (e.g., via older friends), making young contraceptors effectively "hidden" to clinic staff, and compromising the accuracy of clinic-based survey methods. How young females and males procure their methods is not well known and it is assumed their sexual partners, relatives or other adults assist in procurement. Using YRDSS which can effectively allow had to reach population be reached, this study will inform about youth awareness, use and acquisition of family planning methods among both female and male adolescents, and enable reach into a population and topic that may be otherwise hidden. Information from this study will inform programs addressing adolescent and young women to address their sexual and reproductive health needs.

Study limitations

Though RDS is a solid sampling methodology for hard to reach populations, it can be associated with recruitment bias. Some studies have reported challenges with regard to RDS implementation, such as use of paper coupons and confusion around the referral process. Study team will be trained to address such issues.

Study Management:

The YRDSS will be conducted collaboratively between PMA Agile with the International Center for Reproductive Health Kenya (ICRHK). ICRHK is an independent, non-governmental organization (NGO) established in the year 2000.The organization collaborates with the University of Nairobi on intervention projects and research studies with respect to HIV, gender-based violence, family planning, adolescent and youth health, and reproductive health. ICRHK is a World Health Organization (WHO) collaborative centre on reproductive health since 2004

The local principal investigator is responsible for overseeing all in-country implementation, including adherence to study protocols, oversight of data quality assurance, and supervision of fieldwork. JHSPH staff will ensure overall adherence to research plan, provide technical assistance, and ensure all study team members have completed human subject research training. During the first month of data collection, a team member from JHSPH will be on site in country to ensure to appropriate recruitment of participants and data collection process is followed.

The overall study team

Name	Role	Background/Qualifications	Study Responsibilities
Amy Tsui	Principal Investigator and Project Director	Dr. Amy Tsui is a Professor in the Department of Population, Family and Reproductive Health at JHSPH, Senior Scholar in the Bill & Melinda Gates Institute for Population and Reproductive Health, and Principal Investigator for PMA Agile. She has a PhD in Demography from the University of Chicago.	Oversight and management for YRDSS
Michele Decker	Co-Principal Investigator	Dr. Michele Decker is an Associate Professor in the Department of Population, Family and Reproductive Health at JHSPH. She has implemented RDS with a range of high-risk populations including urban youth in multiple settings, female sex workers, and migrant workers at the Thai-Burma border. She has an ScD in Social Epidemiology from Harvard School of Public Health.	Oversight of RDS training, implementation, monitoring and analysis for YRDSS; technical support for AIBEF team
Alain Koffi	Co-Investigator	Dr. Alain Koffi is an Assistant Scientist in the Department of International Health. Prior to that position, Dr. Koffi completed a post-doctoral fellowship at the Gates Institute for Population,	Oversight, management and analysis for YRDSS

		Family and Reproductive Health at the Johns Hopkins Bloomberg School of Public Health. Dr. Koffi was initially trained as a medical doctor at the University of Cocody in Abidjan, Côte d'Ivoire and pursued additional academic training at the Tokyo Medical and Dental University acquiring a PhD in Public Health. Dr. Koffi's research interests reside in human populations, reproductive health, and human health. His work involves survey implementation, data analysis techniques, and research on the biological causes and determinants of maternal, neonatal and child morbidity and mortality	
Peter Gichangi	Principal Investigator-Kenya	Prof. Peter Gichangi is the Country Director of International Centre for Reproductive Health, Kenya. He is the PI for PMA2020 and PMA Agile projects in Kenya. He is an associate Professor University of Nairobi. Prof Gichangi received his PHD from University Nairobi	Local oversight, management and analysis for all YRDSS activities in Kenya. Primary point of contact with JHU on YRDSS survey matters
Mary Thiongo	International Centre for Reproductive Health Kenya	Mary has been managing projects, designing and implementing Monitoring and evaluation for 9+ years with experience on various projects including HIV prevention, care and treatment among key populations, sexual and gender based violence, adolescent and youth program, family planning. She is the Technical advisory for PMA2020/Kenya and the Monitoring and evaluation Manager for ICRHK. She hold a degree in Computer Information Systems from Kenya Methodist University	Local oversight, management and analysis for all YRDSS activities in Kenya. Secondary point of contact with JHU on YRDSS survey matters
Meagan Byrne	Program Specialist	Meagan Byrne is a PMA Agile Program Specialist. She has experience in RDS analysis and study implementation in low-resource settings, most recently with the YRDSS in Abidjan. She has a MSPH from the Johns Hopkins School of	RDS training; technical and programmatic support

		Public Health, with a concentration in sexual and reproductive health.	
Titilope Akinlose	Program Officer	Titilope Akinlose is the PMA Agile Program Officer. She provides administrative, coordination, and technical assistance to the PMA Agile program activities in Baltimore and partner countries. She has an MPH in Health Policy and Administration from the University of Illinois at Chicago.	Coordinating the technical and logistical contributions from JHU to YRDSS data collection being implemented by AIBEF.
Kurt Dreger	Sr. Programmer Analyst	Kurt Dreger is the PMA Agile data engineer. He provides computer and programming assistance to the PMA Agile program in Baltimore and partner countries. He has a BS in Electrical Engineering from the University of Delaware.	Providing technical support for programming surveys, coupons, and other IT components of the study
Jessica Mirano	Program Officer	Jessica Mirano is the M&E Program Officer for The Challenge Initiative (TCI), supporting in the collation, analysis, and reporting of data from various sources. In 2015, she led the conduct of an RDS for an HIV prevalence survey among people who inject drugs in three cities in the Philippines. She has an MPH concentrated in Epidemiology and Biostatistics from JHSPH.	RDS training; technical and programmatic support

Budget

Support for the YRDSS is provided through a PMA Plus grant from the Bill & Melinda Gates Foundation to the Gates Institute/PMA, JHSPH.

Item	Amount (Kshs)
Personnell	13,031,766
Travel	4,144,000.00
Data collectors	2,240,000.00

Equpment	90,000
Other direct costs	2,927,500
Management	3,364,990
Total	25,798,256

Time lines

Anticipated period of data collection, data cleaning and data analysis of 8 months

Activity	March 2019	April 2019	May 2019	June 2019	July 2019	August 2019	September 2019	October 2019
Submission for ethical approval	X							
Engagement with youth	X							
Training of data collector		X						
Setting up data collection sites		X						
Data collection			X	X				
Data analysis					X	X	X	
Sharing of the findings								x

REFERENCES

1. Badowski G, Somera LP, Simsiman B, Lee HR, Cassel K, Yamanaka A, Ren J. The efficacy of respondent-driven sampling for the health assessment of minority populations. Cancer Epidemiol. 2017 Oct;50(Pt B):214-220. doi: 10.1016/j.canep.2017.07.006.
2. Banke-Thomas, O. E., Banke-Thomas, A. O., & Ameh, C. A. (2017). Factors influencing utilization of maternal health services by adolescent mothers in Low-and middle-income countries: a systematic review. *BMC Pregnancy Childbirth, 17*(1), 65. doi:10.1186/s12884-017-1246-3
3. Chandra-Mouli, V., Lane, C., & Wong, S. (2015). What Does Not Work in Adolescent Sexual and Reproductive Health: A Review of Evidence on Interventions Commonly Accepted as Best Practices. *Glob Health Sci Pract, 3*(3), 333-340. doi:10.9745/GHSP-D-15-00126
4. Chandra-Mouli, V., McCarraher, D. R., Phillips, S. J., Williamson, N. E., & Hainsworth, G. (2014). Contraception for adolescents in low and middle income countries: needs, barriers, and access. *Reprod Health, 11*(1), 1. doi:10.1186/1742-4755-11-1
5. Denno, D. M., Hoopes, A. J., & Chandra-Mouli, V. (2015). Effective strategies to provide adolescent sexual and reproductive health services and to increase demand and community support. *J Adolesc Health, 56*(1 Suppl), S22-41. doi:10.1016/j.jadohealth.2014.09.012
6. Godia, P. M., Olenja, J. M., Hofman, J. J., & van den Broek, N. (2014). Young people's perception of sexual and reproductive health services in Kenya. *BMC Health Serv Res, 14*, 172. doi:10.1186/1472-6963-14-172
7. Gile KJ, Handcock MS. Respondent-Driven Sampling: An Assessment of Current Methodology. Sociol Methodol. 2010 Aug;40(1):285-327.
8. Heckathorn DD, 1997 Respondent-driven sampling: a new approach to the study of hidden populations. Soc. Probl. 174–199.
9. Johnston L, Hakim AJ, Dittrich S, Burnett J, Kim E, White RG, 2016. A systematic review of published respondent-driven sampling surveys collecting behavioral and biologic data. AIDS Behav. 1–23.
10. KNBS., MOH/Kenya, M. o., Council/Kenya, N. A. C., Development/Kenya, N. C. f. P. a., Institute, K. M. R., & International, I. (2015). *Kenya Demographic and Health Survey 2014*. Retrieved from Rockville, MD, USA:
11. Liu M, McCann M, Lewis-Michl E, Hwang SA. Respondent driven sampling in a biomonitoring study of refugees from Burma in Buffalo, New York who eat Great Lakes fish. Int J Hyg Environ Health. 2018 Jun;221(5):792-799. doi: 10.1016/j.ijheh.2018.04.014. Epub 2018 Apr 30.
12. Ministry of Health (2015). Kenya National Adolescent Sexual and Reproductive Health Policy. In (pp. Nairobi, Kenya).
13. Montealegre JR, Risser JM, Selwyn BJ, McCurdy SA, Sabin K, 2013 Effectiveness of respondent driven sampling to recruit undocumented Central American immigrant women in Houston, Texas for an HIV behavioral survey. AIDS Behav. 17 (2), 719–727. [PubMed: 22961500]
14. Rosemary, M., & Martin, O. (2008). *Down the drain: counting the costs of teenage pregnancy and school drop out in Kenya*: Center for the study of Adolescents.
15. Townsend L, Johnston LG, Flisher AJ, Mathews C, Zembe Y. Effectiveness of respondent-driven sampling to recruit high risk heterosexual men who have multiple female sexual partners: differences in HIV prevalence and sexual risk behaviours measured at two time points. AIDS Behav. 2010 Dec;14(6):1330-9. doi: 10.1007/s10461-010-9753-5.
16. Volz E, Wejnert C, Cameron C, Spiller M, Barash V, Degani I, Heckathorn DD, 2012 Respondent-Driven Sampling Analysis Tool (RDSAT) Version 7.1. Cornell University, Ithaca, NY.
17. World Health Organization, Regional Office for the Eastern Mediterranean, 2013 Introduction to HIV/AIDS and Sexually Transmitted Infection Surveillance: Module 4: Introduction to Respondent-Driven Sampling. http://www.who.int/iris/handle/10665/116864.
